# Assessing fishery interaction on cetaceans stranded along the Italian coastline between 1986 and 2023

**DOI:** 10.1371/journal.pone.0330441

**Published:** 2025-09-17

**Authors:** Guido Pietroluongo, Michela Podestà, Donatella Belluscio, Enrica Berio, Cristina Canonico, Cristina Casalone, Federica Cavaliere, Cinzia Centelleghe, Luca Ceolotto, Cristiano Cocumelli, Bruno Cozzi, Daniele Denurra, Alessandra Di Donato, Gabriella Di Francesco, Giovanni Di Guardo, Fabio Di Nocera, Ludovica Di Renzo, Stefano Gavaudan, Federica Giorda, Giuseppe Lucifora, Leonardo Marino, Letizia Marsili, Sergio Migliore, Ilaria Pascucci, Gianni Pavan, Antonio Petrella, Antonio Pintore, Roberto Puleio, Silva Rubini, Giuliana Terracciano, Anna Toffan, Carla Grattarola, Sandro Mazzariol

**Affiliations:** 1 Department of Comparative Biomedicine and Food Science, University of Padova, Legnaro, Italy; 2 Consorzio Nazionale Interuniversitario per le Scienze del Mare (CoNISMa), Roma, Italy; 3 Museo Civico di Storia Naturale di Milano, Sezione di Zoologia dei Vertebrati, Milano, Italy; 4 Istituto Zooprofilattico Sperimentale della Puglia e della Basilicata, Foggia, Italy; 5 Department of Prevention, Local Veterinary Services, ASL1 Sistema Sanitario Regione Liguria, Sanremo, Italy; 6 Istituto Zooprofilattico Sperimentale dell’Umbria e delle Marche “Togo Rosati”, Perugia, Italy; 7 Istituto Zooprofilattico Sperimentale del Piemonte, Liguria e Valle d’Aosta, Torino, Italy; 8 National Reference Center for Diagnostic Investigations in Stranded Marine Mammals (C.Re.Di.Ma.), Torino, Italy; 9 Interuniversity Center for Cetacean Research (CIRCE), Genova, Italy; 10 Istituto Zooprofilattico Sperimentale del Lazio e della Toscana, Roma, Italy; 11 Istituto Zooprofilattico Sperimentale della Sardegna, Sassari, Italy; 12 Istituto Zooprofilattico Sperimentale della Lombardia e dell’Emilia Romagna “Bruno Ubertini”, Ferrara, Italy; 13 Istituto Zooprofilattico Sperimentale dell’Abruzzo e del Molise “G. Caporale”, Teramo, Italy; 14 Veterinary Medical Faculty, University of Teramo, Teramo, Italy; 15 Istituto Zooprofilattico Sperimentale del Mezzogiorno, Portici, Italy; 16 Centro Studi Cetacei, Pescara, Italy; 17 Department of Physical Sciences Earth and Environment, University of Siena, Siena, Italy; 18 Istituto Zooprofilattico Sperimentale della Sicilia, Palermo, Italy; 19 Department of Earth and Environment Sciences, University of Pavia, Pavia, Italy; 20 Istituto Zooprofilattico Sperimentale del Lazio e della Toscana, Pisa, Italy; 21 Istituto Zooprofilattico Sperimentale delle Venezie, Legnaro, Italy; University of Messina, ITALY

## Abstract

Monitoring stranded cetaceans represents a strategic method to assess their health, conservation status, and ecological role in the marine ecosystem. Efficient stranding networks and standardized protocols are essential to monitor this phenomenon and investigate its causes. This study assesses the evidence of fishery interaction on stranded cetacean carcasses found along the Italian coastline from 1986 to 2023. Evidence assessment and *post-mortem* investigation methods evolved over three macro-periods, from non-standardized reporting (1986–2014) to an integrated national stranding network (2015–2019), and finally to the creation of a new standardized, evidence-based diagnostic framework under the EU-funded LIFE DELFI project (LIFE18 NAT/IT/000942) (2020–2023). A total of 5355 cases were selected for this analysis. A literature review and evidence of interaction on stranded carcasses supported the categorization of findings, ranging from case history to pathological observations, allowing the assessment of temporal variation, demographic parameters, geographical distribution, and fishing gear identification. Evidence of fishery interaction was found in 12.89% of the cases (690/5355), with an annual average of 18.15 affected animals, and fishery interaction was identified as the likely cause of death in 10.32% of the cases. The most frequently reported species were *Stenella coeruleoalba* and *Tursiops truncatus*, showing significant differences in fishery interactions, particularly in relation to sex, age class, and geographical distribution. Adult male *Tursiops truncatus* exhibited a higher susceptibility to gillnet interaction in the Adriatic Sea. The results of this study emphasize the importance of standardized *post-mortem* investigations and long-term monitoring to identify risk hotspots, implement species- and region-specific mitigation strategies, and establish threshold values for cetacean conservation.

## 1. Introduction

The monitoring of marine mammal strandings may provide insights into the health and conservation status of free-ranging animals in specific marine areas [1]. The collection and analysis of data on marine protected species can also yield information useful for assessing the major anthropogenic threats. It may also support shaping mitigation policies that target specific factors. Moreover, data from stranding events allow for the comparison of spatial and temporal trends. The results consequently contribute to understanding the extent of anthropogenic pressures and support the development of quantitative indicators [2–4]. Finally, monitoring the mortality of these species represents a strategic method to assess trends in mortality patterns, diseases, climate change, and environmental degradation from a One Health perspective [5,6]. Efficient stranding networks are essential to monitor stranding events and investigate their causes. Their success is based on the coordinated efforts of various institutions to track animal carcasses found ashore and conduct standardized *post-mortem* investigations [7,8].

Marine mammals are included in the EU Habitats Directive (1992/43/EC), which protects them from deliberate capture or killing. They are also included in other EU Directives, such as the Marine Strategy Framework Directive (2008/56/EC), which recommends assessing the impact of by-catch and defining the mortality rate per species for long-term viability. Furthermore, it calls on Member States to establish threshold values, through regional or sub-regional cooperation, that ensure sustainable levels which do not threaten the species, and to implement effective management actions.

Due to its central position in the Mediterranean Sea and the growing need to understand the causes of cetacean mortality along its coastline, Italy made its first attempt to establish a data collection system in the late 1970s. Gradually, this volunteer-based effort led to the creation of the first Italian Stranding Network (ISN) in 1986. In 2006, the Italian Ministry of the Environment supported the establishment of an official stranding database to centralize the collection of basic data from stranding events, including location, species, and evidence of human interactions [9]. In 2015, the Ministry of the Environment and the Ministry of Public Health conceived a multiparty institutional plan to study stranding events, with the joint efforts of public veterinary institutes and laboratories, universities, museums, and other governmental agencies [10].

In 2020, the officially recognized ISN adopted the document on best practices for *post-mortem* investigations and tissue sampling in Europe [8] along with the ACCOBAMS Resolution 6.22 [11] to harmonize *post-mortem* investigations as well as the interpretation of evidence of cetacean interaction with human activities. Additionally, as part of the EU-funded project LIFE DELFI (LIFE18 NAT/IT/000942) [12], a diagnostic framework for assessing fishery interaction was developed [13], based on existing literature and supported by *ad hoc* training.

In this context, the present study summarizes data collected by the ISN from the beginning of its work in 1986–2023. The summary focuses on the evidence of fishery interaction on the stranded cetacean carcasses to provide insights into the impact of this anthropogenic threat, including the identification of specific fishing gears, risk hotspots, and the most exposed species. The aim is to improve diagnostic and forensic approaches in support of mathematical models to assess the impact of fishery interaction on marine mammal populations and devise targeted conservation policies.

## 2. Materials and methods

Cetaceans included in this study were found stranded dead on the shores of Italy during 38 years (1986–2023). Data were initially collected and published in the annual bulletin of the Natural History Museum of Milan and, since 2006, on an online national stranding database (Banca Dati Spiaggiamenti – BDS) [14]. For references to the whales and dolphins of the Italian waters, see [15–17]. Within the database, the dataset on cetacean strandings with evidence of fishery interaction was analyzed to assess the average annual stranding rate across three defined macro-periods. Each macro-period was selected to reflect changes in the monitoring efforts to better evaluate potential anthropogenic pressures and environmental factors over time. Besides information on the stranded specimens (dead vs alive, species, sex, age class, decomposition code of the carcass – DCC, nutritional condition code – NCC) as well as the stranding events (date, location, number of animals, institution involved in the data collection), the BDS also includes notes from the operator describing additional and relevant details. These notes provide valuable insights into the event, possible causes of death (COD), and relevant evidence suggesting interactions with human activities.

### 2.1 Stranding data and macro-period approach

In the 38 years of study, 6393 cetaceans found stranded along the 8000 km of the Italian coastline were reported in the BDS. Records reporting live animals or cetaceans with poor or incomplete information, as well as stranding reports lacking information, were not included in the present study. As a result of this selection, a total of 5355 carcass reports were considered.

During the 38 years, *post-mortem* investigations were carried out with different approaches [18,19] by different personnel with different skills and expertise. To harmonize data and the approaches for the data collection, as stated above, three relevant macro-periods were identified according to the tiered approach proposed by IJsseldijk et al. (2019) [8]:

1. Period 1 (P1): from 1986 to 2014, basic data from external examinations were collected on a voluntary and occasional basis, without standardized procedures, and by operators lacking specific training in *post-mortem* examinations;
2. Period 2 (P2): from 2015 to 2019; public veterinary services and laboratories have been integrated into the Italian Stranding Network-ISN with centralized coordination, collecting samples for monitoring infectious diseases and evidence of human interaction. Furthermore, *post-mortem* findings collected since 2015 have been published in official annual reports by the National Reference Center for Diagnostic Investigations in Stranded Mammal Diseases (Centro di Referenza per le Indagini Diagnostiche sui Mammiferi marini spiaggiati – C.Re.Di.Ma.). During this period, necropsies were routinely performed to hypothesize the COD and any human interaction. However, the interpretation of *post-mortem* findings was often not harmonized along the entire Italian coastline between 2015 and 2019.
3. Period 3 (P3): from 2020 to 2023; the adoption of the standardized diagnostic frameworks under the EU-funded LIFE DELFI project (LIFE18 NAT/IT/000942) allowed training of the Italian pathologists. This LIFE DELFI framework, specific to fishery-interaction evidence, facilitated the interpretation of *post-mortem* findings, relying on the most up-to-date and peer-reviewed literature.

Data from all three macro-periods were used for a retrospective analysis of fishery interaction evidence (Tier 1 and 2 according to IJsseldijk et al., 2019) [8]. In period P2 and P3, fishery interaction as COD was investigated (Tier 3 according to IJsseldijk et al., 2019) [8].

### 2.2 Retrospective review and analysis of fishery-interaction evidence (P1, P2, and P3)

For all the periods, the fishery-related findings were categorized according to Moore et al. (2013) [20]. In all cases, data collection is primarily focused on the stranding event (date, location, and Geographical Sub Areas - GSAs, established from the FAO/GFCM/33/2009/2 Resolution), biometric data (species, sex, age class), as well as the decomposition condition code (DCC) and nutritional condition code (NCC) of the carcass. The categories of evidence of fishery interaction are summarized in Table 1.

**Table 1:** Data and fishery-interaction evidence categories for the data analysis (P1, P2, P3). The table is organized in “General categories”, “Specific categories”, and their “Description”.

GENERAL CATEGORIES	SPECIFIC CATEGORIES	DESCRIPTION
Stranding data	Location	Region and GSA
	Date	Year
	Necropsy	Yes/No
Carcass identification	Species	All the species; ND was also counted and, depending on the individual characteristics, was categorized as small or large cetaceans when possible
	Age category	Adult or juvenile (newborn, calf, sub-adult)
	Sex	Male (M) and female (F)
	DCC	1-5
	NCC	1-5
Certain evidence of interaction with fishing activities	By-catch in animal history	Report of the lethal event while fishing or the landing of the carcass at the harbor by fishermen, observers, or authorities
	Presence of fishing gear	Presence of nets, lines, ropes, or hooks entangling external parts of the carcass
	Larynx entanglement	Presence of net, net remains, fishing gears, and lines entangling the larynx
	Ingestion of fishing gear	Presence of net, net remains, fishing gears and lines, hooks in the gastric chambers
Physical injuries directly related to an interaction with fishing activities	Net marks	Fresh linear skin lesions and furrows in or around the mouth, fin, flipper, or tail, or encircling one or more extremities
	Sharp wounds and amputation	Amputation of fins, flippers, flukes, or tails; cuts on the edge of the mouth, fin, flippers, or tail; body slits and incisions
	Penetrating wounds	Penetrating injuries likely caused by stabs, harpoons, gunshots, etc.

Based on the evidence and the physical injuries directly related to fishery interaction, the manner of interaction was categorized as follows [21,22]:

a) Accidental interaction: it includes all the evidence suggesting an incidental interaction between an active gear and the animal independently from its behavior (i.e., by-catch in animal history, presence of fishing gear, net marks);
b) Behavior-associated interaction: it includes all the evidence clearly resulting from foraging behavior on gillnets (i.e., larynx entanglement, ingestion);
c) Non-accidental interaction: it includes all the *post-mortem* findings indicating a deliberate action of the fishermen on the animal found (alive or dead) in or close to any fishing gear (i.e., sharp wound, amputation, penetrating wound).

The data analysis also focused on temporal trends and spatial distributions in the GSAs.

### 2.3 Fishery interaction as COD (P2, P3)

Between 2015 and 2023 (P2, P3), the Italian Stranding Network-ISN conducted necropsies and *post-mortem* investigations on 790 carcasses out of 1772 (44.58%: n = 790/1772) reported in the national stranding database-BDS (P2: n = 469/1038; P3: n = 321/734) according to standardized protocols [11]. The presence of any infectious and parasitic diseases was assessed according to Grattarola et al. (2024) [10]. Since 2020 (P3), fishery interaction evidence has been assessed according to the LIFE DELFI framework. The framework [13] was developed from a review of the most recent peer-reviewed literature, including categories summarized in Table 2. The categories were used by pathologists to support their interpretation of *post-mortem* evidence combining the different data including gross, microscopic and ancillary examinations. When a single “Confirmed” evidence of anthropogenic trauma was observed, the COD was certainly identified as fishery-related. On the opposite, when only informative findings were reported, the COD was categorized as “Probable” or “Suspect” and not included in our results as related to fishery.

**Table 2:** Summary of the categories of the evidence of fishery interaction included in the LIFE DELFI framework, along with their relevant literature.

CATEGORY OF FINDINGS	Description	Finding rates to define the COD according to Moore et al. (2013)	REFERENCES
History	Availability of reliable direct or indirect information indicating that the animal was accidentally captured in fishing operations (by-catch), including eyewitness accounts, photographic documentation, fisher statements, or retrieval of the animal directly from fishing gear	Confirmed	[20,23,24]
Fishing gear presence	Entanglement in fishing gear, presence of fishing gears occluding the esophagus/stomachs	Confirmed	[20,23–25]
Physical injuries directly related to the interactions	Gross findings: net marks, bulging eyes/hyphema, separation of the rectus abdominis muscle, *linea alba* erniation Histopathological findings: disseminated gas bubbles, multiorgan congestion	Confirmed	[20,23,24,26,27]
Physical injuries indirectly related to the interactions	Gross findings: sharp wounds, amputation, penetrating wounds, contusions, fractures Histopathological findings: capture myopathy	Probable	[20,23,24,27]
Non-specific pathological findings	Histopathological findings: microscopic muscular hemorrhages, pulmonary and vascular changes	Suspect	[24,26,27]
Information on recent feeding	Presence/absence of recently ingested esophagic/gastric content, good/poor NCC	Probable	[20,23]
Absence of other possible causes of death	Evidence from the necropsy and ancillary analyses does not indicate other COD, such as ongoing natural diseases (e.g., infectious diseases, age-related mortalities, neoplasms, etc.) or anthropogenic factors (e.g., vessel strikes, acoustic-related barotrauma, etc.).	Suspect	[10,23,24,26]

In P2 and P3, *post-mortem* findings support the identification of specific fishing gear interaction as the CODs, which were categorized as follows: a) peracute underwater entrapment (PUE) [20,22,27] suggested an incidental catch in non-static nets; b) the entanglement of the body and/or larynx in passive nets, along with the ingestion of a significant amount of net/net fragments suggested interaction with static nets as gillnets [21,28]; c) chronic lesions suggested the interaction with ghost nest, driftnet, fishing lines, and ropes entangling the body extremities and leading the animal to death due to the injuries and/or starvation [20,28].

### 2.4 Statistical analysis

Statistical analyses focused primarily on the most frequently represented species in the dataset (*Tursiops truncatus* and *Stenella coeruleoalba*), examining potential differences in the occurrence of fishery interactions by age class, sex, and geographical distribution (GSA). Additional analyses assessed temporal trends across three macro-periods of the following specific indicators of interaction (i.e., presence of fishing gear, net marks, ingestion, entanglement, traumatic lesions) between species, in order to identify species-specific patterns potentially linked to behavior or exposure risk. Data processing and exploration were conducted in Python [29]. Depending on the nature of the variable, different statistical methods were employed to assess relationships among variables. The Kruskal-Wallis test [30] was used for numeric variables across multiple groups, followed by *post-hoc* pairwise comparisons via Dunn’s test [31]. The Chi-Square test was used to analyze categorical variables [32]. Additionally, a categorical logistic regression was performed, which required recording categorical variables into binary form. Statistical significance was set at a p-value threshold of <0.01 [33], indicating that associations with p-values below this threshold were considered statistically significant. This rigorous approach ensured that only robust relationships were included in the analysis.

## 3. Results

Between 1986 and 2023, 6393 cetaceans were found stranded along the Italian coastline, with an average of 168.23 animals/year. Among the 5355 cases selected for the completeness of the collected data, evidence of fishery interaction was reported on 690 carcasses, accounting for 12.89% of the selected cases (690/5355) with an annual average of 18.15 animals per year showing signs of interaction with fisheries. The analysis and illustrations of the results are based only on these 690 specimens. These carcasses belonged to 4 prominent families, namely 560 *Delphinidae*, 50 *Physeteridae*, 11 *Ziphiidae*, and 9 *Balaenopteridae*. Due to logistical constraints and/or DCC limitations, it was not possible to report the species in the national stranding database-BDS for 60 carcasses. The most frequently recorded species with evidence of fishery interaction (n = 521/690) were *Stenella coeruleoalba* (Sc) and *Tursiops truncatus* (Tt). Among the other species described in supplementary materials (S1 Table), it is noteworthy that 50 *Physeter macrocephalus* (Pm) showed evidence of fishery interaction, with 36 cases of entanglement in fishing gear. Driftnets were identified in 9 cases between 1990 and 1995, and in 2 additional cases in 2017.

### 3.1 Temporal variation in evidence of fishery interaction (P1, P2, P3)

The results of the temporal variation show a decreasing trend in the prevalence of stranded animals with evidence of fishery interaction. During P1, the average rate of animals showing interaction with fisheries was 19.24 (min = 1; max = 59) cetaceans per year, based on a total of 558 cases out of 4493 stranded animals (12.42%; CI 95%: 11.46–13.40) over 29 years. P2 exhibited a similar average yearly rate of 20.75 (min = 13; max = 22) animals, showing findings suggesting interaction with fishing activities, with 83 cases out of the 481 stranded animals (17.23%; CI 95%: 13.93–20.79) in the 4 years. Finally, P3 presented the lowest number of animals per year (12.25; min = 6; max = 19) with clear evidence of interaction with fishing activities, based on 49 cases on the 381 stranded animals (12.86%; CI 95%: 9.71–16.27) over 4 years.

**Fig 1:**
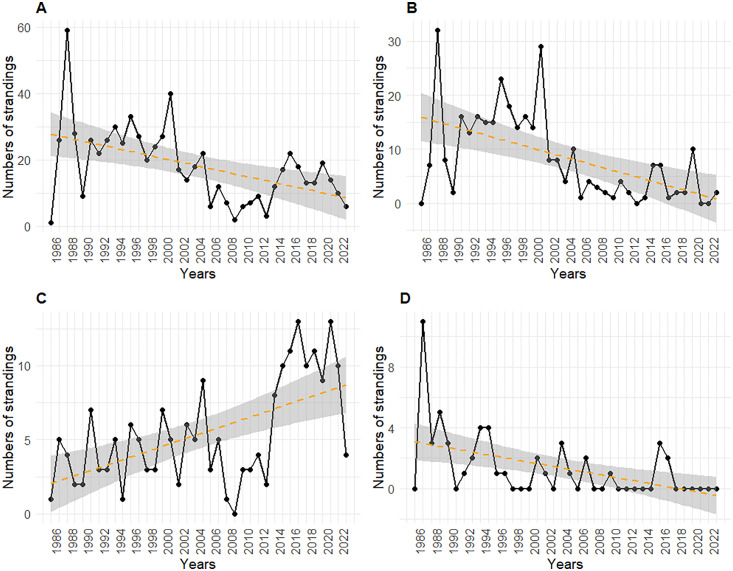
Temporal trends of cetacean strandings with evidence of fishery interaction from 1986 to 2023. (A) Total strandings; (B) *Stenella coeruleoalba* (Sc); (C) *Tursiops truncatus* (Tt); (D) *Physeter macrocephalus* (Pm).

The analysis revealed clear differences in fishery interaction rates across the three macro-periods. A progressive decrease was observed, with the lowest values recorded in the most recent period (P3). This overall downward trend is also illustrated in Fig 1A. Despite this general pattern, interspecific comparisons indicate that the temporal dynamics of interactions with fishing activities vary between species.

Focusing on the two main represented species with evidence of fishery interaction within the three macro-periods, *Stenella coeruleoalba* (Sc) accounted for a higher proportion of the total carcasses (n = 317) compared to *Tursiops truncatus* (Tt) (n = 204). Sc was more represented in P1, showing three major peaks in 1988 (n = 32), 1996 (n = 23), and 2001 (n = 29). Its proportion decreased over time, highlighting a shifting pattern in species distribution (Fig. 1B). Tt was consistently reported across the three periods, with no clear peaks but a tendency to be more represented than Sc after 2013, particularly with a steady increase in numbers (Fig. 1C). Regarding *Physeter macrocephalus* (Pm), its presence within fishery interaction cases was lower than Sc and Tt (n = 50), with a clear decline over time. However, a notable peak was observed in 1987 (n = 11), after which numbers steadily declined. This suggests a potential shift in interactions with fisheries or changes in reporting and detection rates over the years (Fig. 1D) (statistical results are shown in Table 3).

**Table 3:** Summary of statistical result of temporal variation in evidence of fishery interaction (only significant correlations are shown).

Statistical Test	Statistic
General differences in fishery interaction rates across P1, P2 and P3	H-test = 37.0, (df = 2), p < 0.01
Differences in fishery interaction rates between P1 and P2	p < 0.01
Differences in fishery interaction rates between P1 and P3	p < 0.01
Differences in fishery interaction rates between P2 and P3	p = 0.0072
Linear regression – General decreasing trend in fishery interaction	m = −0.518; R² = 0.2476
Differences in species distribution between Sc and Tt	χ² = 83.96; p < 0.01
Linear regression – Decreasing trend of Sc over time	m = −0.4087; R² = 0.3082
Linear regression – Increasing trend of Tt over time	m = 0.1805; R² = 0.3232
Linear regression – Decreasing trend of Pm over time	m = −0.0952; R² = 0.2433

### 3.2 Fishery interaction evidence in relation to age class and sex (P1, P2, P3)

In 68.3% of carcasses with fishery interaction (n = 471/690), the age class was not assessed. When age class was estimated (n = 219), juveniles were less represented than adults (n = 79 vs. 140). For Tt, 74 were adults, 26 juveniles/newborns, and 104 had undetermined ages. For Sc, 39 were adults, 41 juveniles/newborns, and 237 had an undetermined age.

**Fig 2:**
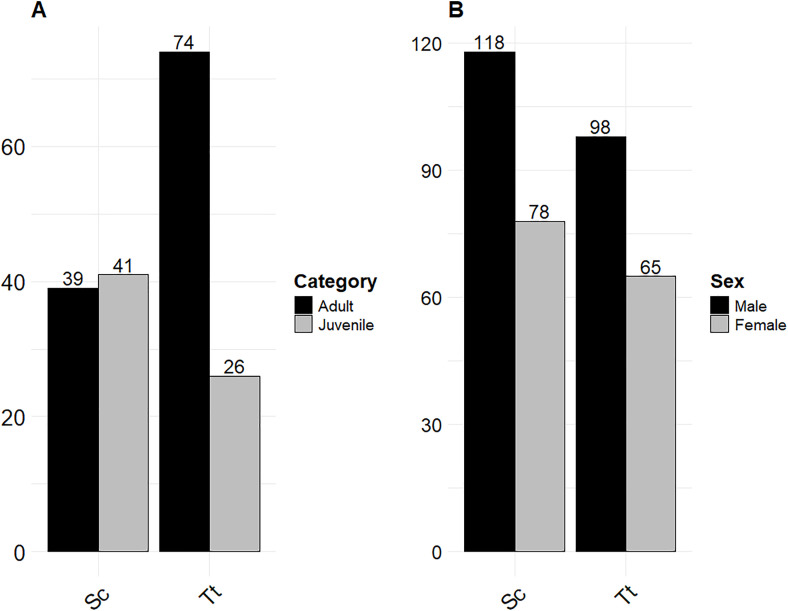
Age class (A) and sex (B) prevalence in *Stenella coeruleoalba* (Sc) and *Tursiops truncatus* (Tt) with evidence of fishery interaction between 1986 and 2023.

Adult *Tursiops truncatus* (Tt) showed a higher probability of fishery interaction compared to juveniles. In contrast, no apparent difference was observed between age classes in *Stenella coeruleoalba* (Sc). When comparing species, adult Tt were more affected than adult Sc, while no difference was noted between juvenile individuals. These findings highlight a species-specific and age-related pattern in fishery interactions (Fig. 2A), with adult Tt appearing particularly vulnerable. Considering the sex for the total carcasses with fishery interaction (Fig. 2B), males (n = 246) were more represented compared to females (n = 162), while in 282 carcasses, it was not possible to determine the sex due to the advanced DCC.

In *Tursiops truncatus* (Tt), males were more frequently involved in fishery interactions compared to females. A similar pattern was observed in *Stenella coeruleoalba* (Sc), where males were also more affected than females. When comparing species, male Tt were more impacted than male Sc, while no substantial difference was observed between females of the two species. These findings indicate a sex- and species-specific pattern, with male Tt being particularly vulnerable (statistical results are shown in Table 4).

**Table 4:** Summary of statistical results of Fishery interaction evidence in relation to age class and sex (only significant correlations are shown).

Statistical Test	Statistic
Fishery interaction - Adult (Tt) vs. Juvenile (Tt)	χ² = 29.26; p < 0.01
Fishery interaction - Adult (Tt) vs. Adult (Sc)	χ² = 40.59; p < 0.01
Fishery interaction – Male (Tt) vs. Female (Tt)	χ² = 10.46; p = 0.0012
Fishery interaction – Male (Sc) vs. Female (Sc)	χ² = 11.23; p = 0.0008

### 3.3 Analysis of the prevalence of *post-mortem* evidence of fishery interaction (P1, P2, P3)

**Fig 3:**
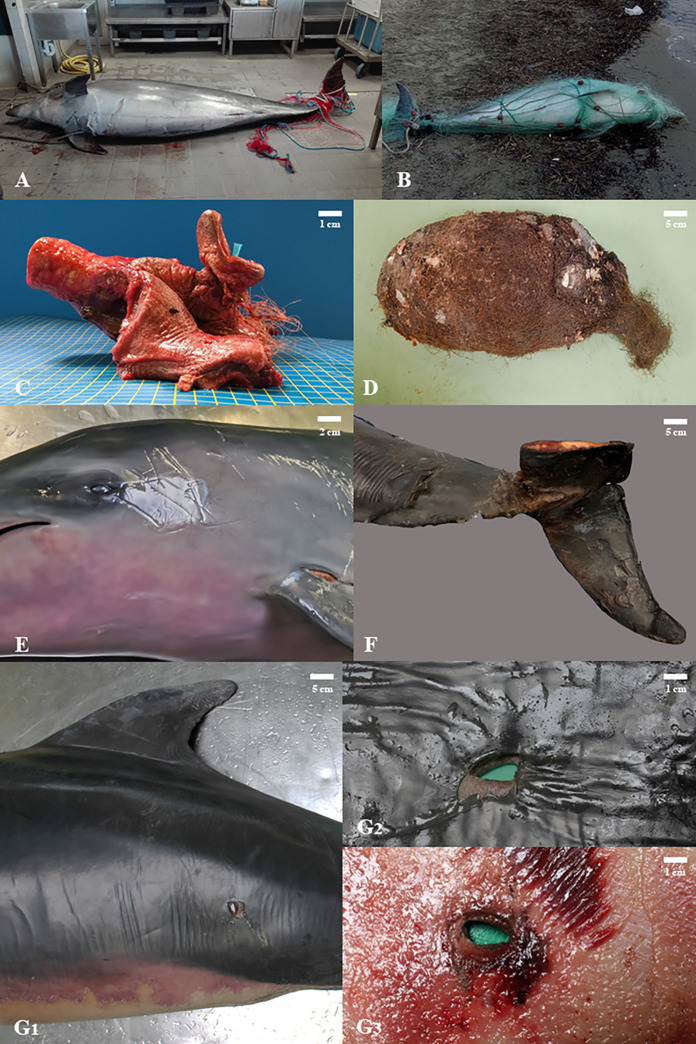
Evidence of fishery interaction in cetaceans stranded along the Italian coastline: A. By-catch in animal history; B. Presence of fishing gears; C. Larynx entanglement; D. Ingestion; E. Net marks; F. Amputation; G. Penetrating wounds: G1. External view of the penetrating wound; G2. Focus on the penetrating wound; G3. Internal focus of the penetrating wound.

Fig 3 summarizes the most reported *post-mortem* evidence. During P1, the most frequently reported category was “Intentional interaction”, with 311 cases, accounting for 6.92% of the total strandings (n = 331/4493). “Unintentional interaction” also represented a significant finding, with 268 cases (5.96%), while net-foraging behavior was absent. In P2, “Intentional interaction” remained relevant in 40 cases (8.32%), “Unintentional interaction” decreased to 28 cases (5.82% of 481 total strandings), while “Net-foraging behavior” increased, reaching 39 cases (8.11%). In P3, “Intentional interaction” showed a significant drop to 13 cases (3.41%), “Unintentional interaction” rose to 26 cases (6.82% of 381 total strandings), and “Net-foraging behavior” decreased to 17 cases (4.46%).

**Fig 4:**
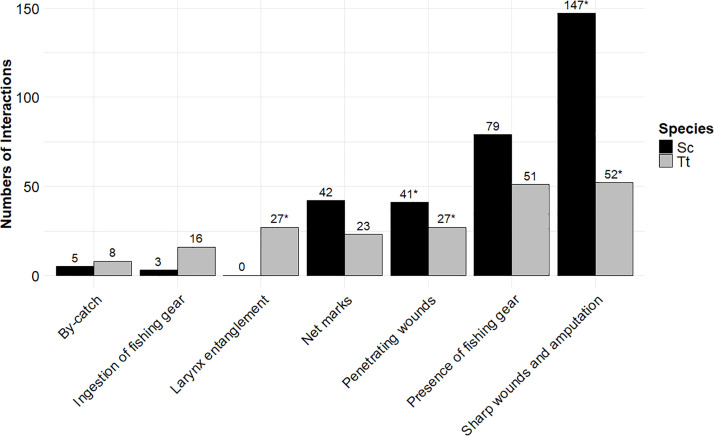
Distribution of anthropogenic interaction categories for *Stenella coeruleoalba* (Sc) and *Tursiops truncatus* (Tt). Bars represent the number of specimens per category, asterisks (*) indicate cases where multiple interaction signs were observed in the same specimen (not represented in the graph).

The analysis of findings suggesting a fishery interaction as described in Table 1 is summarized considering the entire study period. Fig. 4 shows the data on fishery interactions observed in Sc and Tt. Multiple interaction signs were recorded in the same specimen (S1 Table). Specifically, “Sharp wounds” and “Amputation” were associated with “Net marks” (6 Sc and 2 Tt), “Fishing gear entanglement” (5 Sc and 3 Tt), and “Larynx entanglement” (1 Tt). “Penetrating wounds” were associated with “Net marks” (2 Sc and 1 Tt), “Fishing gear entanglement” (1 Sc and 2 Tt), and “Larynx entanglement” (1 Tt).

The category “By-catch in the animal history” was reported at similar frequencies in both *Stenella coeruleoalba* (Sc) and *Tursiops truncatus* (Tt). Likewise, the presence of fishing gear and net marks showed no substantial differences between the two species. In contrast, larynx entanglement was observed only in Tt, indicating a marked species-specific pattern. Ingestion of fishing gear was also more frequently documented in Tt, suggesting differences in exposure or foraging strategies between species. As for traumatic injuries, sharp wounds and amputations were more prevalent in Sc, while penetrating wounds appeared equally distributed between the two species (statistical results are shown in Table 5).

**Table 5:** Summary of statistical results of the prevalence of post-mortem evidence in fishery interaction (only significant correlations are shown).

Statistical Test	Statistic
Larynx entanglement observation	OR=0.01; p < 0.01
Ingestion of fishing gear observation	OR=0.11; p < 0.01
Sharp wounds and amputation observation	OR=2.56; p < 0.01

### 3.4 Fishery interaction evidence distribution in GSAs (P1, P2, P3)

Cetacean carcasses with evidence of fishery interaction were reported in all the GSAs. In particular, GSA 10 (n = 196), 9 (n = 141), 19 (n = 106), 11 (n = 100), and 17 (n = 91) recorded the majority of cases, while only a few cases were reported in GSA 18 (n = 36) and 16 (n = 20).

Differences in fishery interaction patterns between *Tursiops truncatus* (Tt) and *Stenella coeruleoalba* (Sc) were evident across specific GSAs. Sc was more frequently reported in GSA 10 and GSA 19, whereas Tt showed higher involvement in GSA 17 and GSA 18. No notable differences between the two species were observed in GSA 9, GSA 11, and GSA 16 (statistical results are shown in Table 6).

**Table 6:** Summary of statistical results of Fishery interaction evidence distribution in GSAs (only significant correlations are shown).

Statistical Test	Statistic
Differences in fishery interaction patterns – GSA 10 (Tt vs Sc)	χ² = 46.26; p < 0.01
Differences in fishery interaction patterns – GSA 17 (Tt vs Sc)	χ² = 135.87; p < 0.01
Differences in fishery interaction patterns – GSA 18 (Tt vs Sc)	χ² = 8.92; p = 0.0028
Differences in fishery interaction patterns – GSA 19 (Tt vs Sc)	χ² = 41.49; p < 0.01

**Fig 5:**
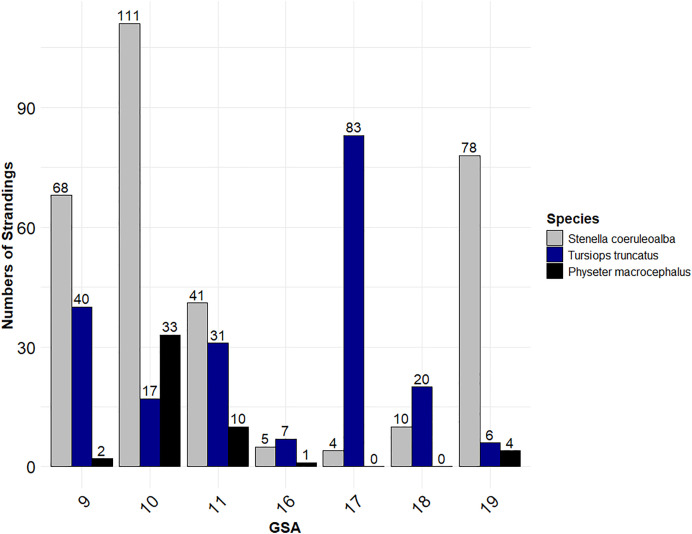
Stranding distribution of *Stenella coeruleoalba* (Sc), *Tursiops truncatus* (Tt), and *Physeter macrocephalus* (Pm) across the GSAs.

Fig. 5 illustrates the distribution of fishery interactions across the GSAs. Pm is included alongside the most represented species (Sc and Tt) to highlight the areas where this endangered species is most at risk.

**Fig 6:**
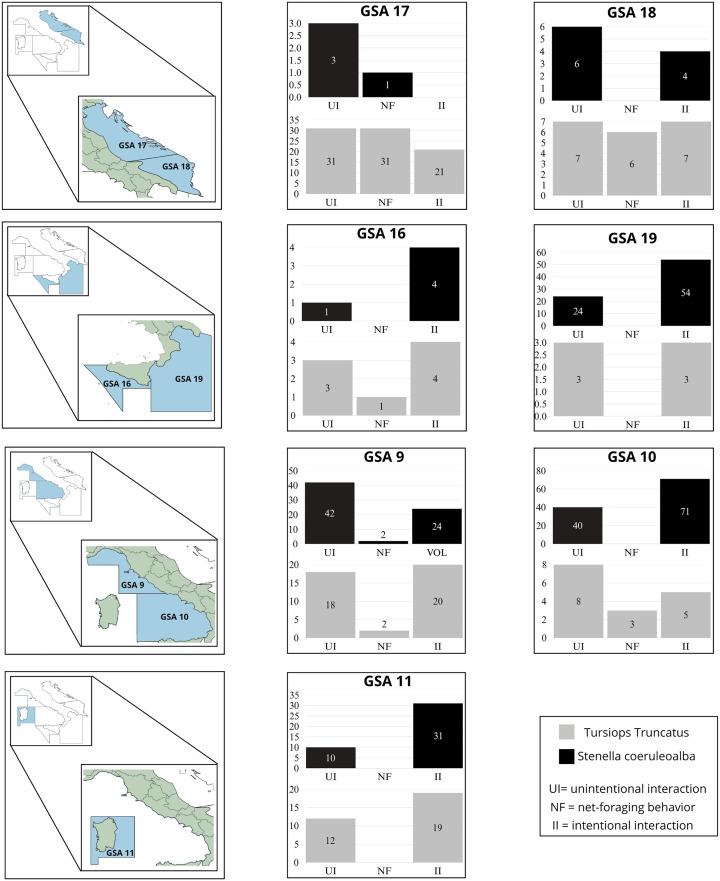
Cetacean-fishery interactions across different GSAs. **The maps show the locations of the analyzed areas, while the bar charts display the number of interactions categorized as “Unintentional interaction”, “Net-foraging behavior”, and “Intentional interaction” for the two most represented species: *Tursiops truncatus* (Tt, in gray) and *Stenella coeruleoalba* (Sc, in black**).

Focusing on significant differences in cetacean-fishery interactions, in GSA 9, Sc shows a high number of unintentional interactions (n = 42) and intentional interactions (n = 24), while Tt has a more balanced distribution, with 18 unintentional interactions, 2 cases of net-foraging behavior, and 20 intentional interactions. In GSA 10, Sc exhibits the highest number of intentional interactions (n = 71), far surpassing Tt, which records 40 unintentional interactions and only 5 intentional interactions. In GSA 11, Sc reaches 31 intentional interactions, whereas Tt is more evenly distributed across the three interaction types, with 12 unintentional interactions, 2 cases of net-foraging behavior, and 19 intentional interactions. In GSA a 16, the number of interactions is relatively low, but Sc still dominates in intentional interactions (n = 4), while Tt is more evenly spread across unintentional interactions (n = 3), net-foraging behavior (n = 1), and intentional interactions (n = 4). In GSA 17, Sc shows a low presence in intentional interactions (n = 1) and unintentional interactions (3), while Tt has a higher and more even distribution with 31 unintentional interactions, 31 cases of net-foraging behavior, and 21 intentional interactions. In GSA 18, Sc is more engaged in net-foraging behavior (n = 6) and intentional interactions (n = 4), while Tt maintains a more balanced presence across unintentional interactions (n = 7), net-foraging behavior (n = 6), and intentional interactions (n = 7). Finally, in GSA 19, Sc shows the second-highest number of intentional interactions (n = 54) and also a significant presence in net-foraging behavior (n = 24), while Tt records only 3 cases in each category. These data are illustrated in Fig. 6.

### 3.5 Fishery-related mortality (P2, P3)

As stated above, the assessment of the fishery interaction as the likely COD was possible during the periods P2 and P3 (2015–2023) on 790 carcasses out of 1772 (44.58%) reported in the national stranding database-BDS. Based on the evidence observed during the *post-mortem* investigations (S2 Table), fishery interaction was deemed as the likely COD in 82 cases (10.32%), respectively 54 in P2 and 28 in P3. Most specimens were Tt (n = 64), followed by Sc (n = 12). Other species involved were 2 *Delphinus delphis*, 1 *Grampus griseus* (Gg), 1 *Globicephala melas* (Gm), and 2 Pm. Adults (n = 47) were more involved compared to young animals (n = 30), as well as males (n = 50) compared to females (n = 28).

Interaction with gillnets was hypothesized as the likely COD in 46 animals (56% of COD due to fishery interaction). In detail, entanglement in gillnet was hypothesized as the likely COD in 12 cases, involving adult Tt, except 1 Sc. Larynx entanglement was hypothesized as the likely COD in 27 Tt, while gillnet ingestion led to death in 5 Tt. Additionally, chronic entanglements in a gillnet as the likely COD were reported in 1 Gm and 1 Dd.

PUE was diagnosed in 16 animals, suggesting by-catch in trawling as the likely COD (19.51% of COD due to fishery interaction). Among these cases, underlying pathological conditions were identified as a predisposing factor in 3 cases (S2 Table).

3 cetaceans (3.66% of the COD due to fishery interaction) were found chronically entangled in driftnet (n = 2 Pm) or fishing line (n = 1 Tt).

Finally, even if *post-mortem* evidence cannot allow the identification of a specific gear, evidence of intentional injuries suggested fishery interactions as the likely COD in 17 cases (20.73% of the COD due to fishery interaction): 8 Sc, 7 Tt, 1 Gg, and 1 Dd.

Interestingly, the distribution of the COD categories within the GSAs showed that fishing net ingestion (n = 4/5) and larynx entanglement (n = 22/27) were mostly reported in GSA 17.

**Fig 7:**
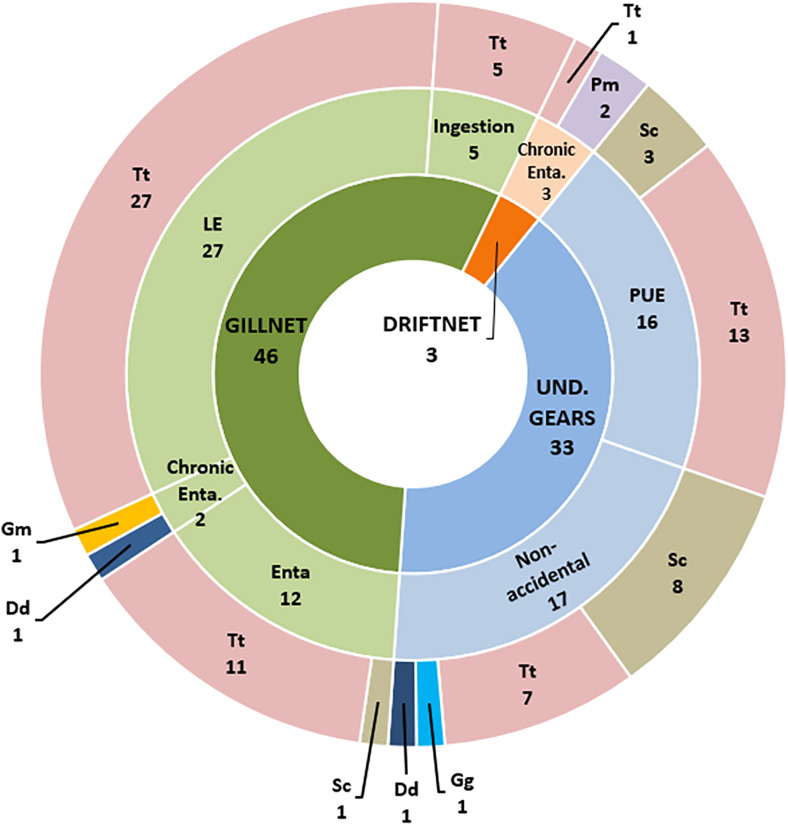
Illustration of the fishery interaction categories associated with the COD. **The innermost ring represents the main categories, including gillnet (GILLNET), undetermined gears (UND. GEARS), and driftnet or fishing line (DRIFTNET).** The middle ring provides a more detailed classification of specific causes, such as entanglement in fishing gear (Enta), larynx Entanglement (LE), chronic entanglement in fishing gear (Chronic Enta.), ingestion (Ingestion), peracute underwater entrapment (PUE), and non-accidental. The outermost ring represents the species involved, identified by their abbreviations: *Stenella coeruleoalba* (Sc), *Tursiops truncatus* (Tt), *Grampus griseus* (Gg), *Delphinus delphis* (Dd), *Globicephala melas* (Gm), *Physeter macrocephalus* (Pm).

Fig. 7 illustrates all the cases of fishery-related mortality. S2 Table summarizes the information on the cases of fishery-related mortality. Due to the small number of specimens, no statistically significant results were found.

## 4. Discussion

The present study summarizes data on the possible interactions between fishery and cetacean strandings along the Italian coastlines between 1986 and 2023. Throughout these 38 years, cetacean stranding monitoring in Italy has faced limitations due to independent methodologies adopted by each institution in the network, resulting in uneven spatial and temporal sampling effort. During P1 strandings, reporting was conducted by volunteer institutions that based their efforts on private resources or limited projects with a special focus on specific areas or threats, depending on their expertise. A variable trend is highlighted, with a progressive decrease in the cases between 1986 and 2014 and a limited number of *post-mortem* investigations [34,35]. After an unusual mortality event of sperm whales in 2009 [36] and an outbreak associated to Cetacean Morbillivirus infection in 2013 [37], in P2 a systemic involvement of public veterinary laboratories helped to cover the entire national coastline ensuring a constant carcass recovery and routine necropsies on stranded animals [10] which has been standardized with the present study (P3). These changes are reflected in the differences in the collected data, with fishery interaction prevalence variation from 12.42% in P1 to 17.23% in P2, and 12.86% in the last period P3. In P1 some of the cases were probably misinterpreted by the lack of standardized *post-mortem* investigations, as in the case of larynx entanglement and ingestion reported for the first time in P2, or in the progressive reduction in penetrating wounds suggesting and intentional killing, likely associated to the lack of skills and expertise of the volunteers involved in reporting in P1. The lack of experience could have led to overreporting, while the use of stranding reports in the historical data review can underestimate the impact of fatalities related to fishery interaction. Penetrating wounds, likely related to deliberate actions by fishermen, progressively decreased over the three periods, in particular in P3 when the LIFE DELFI protocol has been applied with proper training relating most of these *post-mortem* findings to scavenging [20]. It is also worth noting that the number of fishery interactions per year remained similar in P1 and P2 (19.24% and 20.75%, respectively), with a progressive decrease in P3 (12.25%). These variations could reflect not only a real reduction in interactions but also differences in monitoring efforts and tiered approaches in *post-mortem* investigations, as previously noted. Additionally, several other factors may affect carcass stranding rates, including local coastal morphology, sea currents, wind patterns, and the higher probability of strandings of by-caught carcasses compared to those that died of natural causes. The progressive and significant reduction of “undetermined” species, advancements in necropsy approaches, and the harmonization in evidence interpretation and final diagnosis may also explain these fluctuations. Drifting and other computational models can empower the understanding of this phenomenon and the identification of regional and temporal hotspots, providing useful insights to address targeted regional and demographic issues [38].

Despite the limitations, fishery interaction was reported in 12.89% of the examined carcasses found along the Italian coastline, with an average of 18.25 animals per year, and fishery was identified as the likely cause of death (COD) in 10.32% of cases. This decline correlates with an overall decrease in stranding trends but may also indicate that the overall impact of fisheries on cetacean strandings has lessened over time due to various factors. This prevalence is higher than that reported in similar studies in the Western Mediterranean [39] and in the Canary Islands [24], but significantly lower than findings from Croatian waters for a similar period [28].

Stranding monitoring remains a crucial method for monitoring the effective implementation of legal frameworks, such as the EU Habitat Directive (92/43/EEC) and the enforcement of the ACCOBAMS Agreement in 2005, along with the strengthening of monitoring programs and conservation efforts, which supported numerous mitigation initiatives across different areas of the Mediterranean Sea. Additionally, the ban on driftnets in 2002 [40] may have played a major role, as indicated by the progressive reduction of Pm showing impacts from these gears. Despite this clear improvement, fatalities are still occasionally reported in specific areas on this endangered species, such as in the Southern Tyrrhenian Sea (S1 Table), where 2 Pm were observed offshore while swimming entangled in a large-scale driftnet in 2020 [41].

The reduction in fishery interactions over the 38 years may also be due to the significant decline in landings during the same period [42], particularly after 2020. This decline is likely linked to the effects of the SARS-CoV-2 pandemic on human activities, including restrictions during lockdown periods [43,44]. These observations can be further supported by future analysis and comparison of collected data with population density and abundance estimates to assess potential correlations between free-ranging populations and strandings.

A more comprehensive understanding can be gained from data on the most represented species. A significant number of strandings reports by port authorities still indicate an undetermined species or identify the specimen as a “common dolphin”. This latter definition does not indicate an individual *Delphinus delphis*, but simply a small cetacean that may be easily recognized as a delphinid. The increasing dissemination of in-depth knowledge of cetacean systematics among members of strandings networks may help reduce misidentification of specimens and potentially lead to the detection of species previously considered vagrant or rare.

The results revealed significant differences in fishery interactions between Tt and Sc, particularly concerning sex, age class, and GSA. The higher prevalence of adults among Tt carcasses compared to Sc suggests species-specific differences in behavior, habitat use, and/or exposure to fishing activities. Furthermore, while Sc has followed the general trend of a progressive reduction in strandings showing evidence of fishery interaction, Tt is the only species with increasing numbers, particularly during P2 and P3. This species is highly present in GSA 17, which includes the Northern and Central Adriatic Sea [45,46]. Strandings in this area have been consistently monitored since 2002 with the establishment of the Mediterranean Marine Mammal Tissue Bank at the University of Padova [47], confirming the reliability of the collected data.

While no significant differences in abundance across years have been reported in this area, some studies have documented considerable monthly fluctuations affecting interactions [48]. Possible changes in feeding behavior could also be responsible for these positive trends, with fishing net-foraging being the most commonly reported behavior by fishermen in the area [49–51]. The significant dominance of net-foraging interactions, such as larynx entanglement and ingestion, further supports the notion of distinct vulnerabilities. These findings align with previous studies that indicated that Tt is more coastal, interacts more frequently with human activities, and thus may face greater risks from certain types of fishing gear [48]. Furthermore, the inshore habitat preferentially occupied by Tt makes individuals of this species more prone to accumulating and biomagnifying a large number of persistent environmental pollutants in their body tissues [52]. This, in turn, can lead to immunotoxic, neurotoxic, and endocrine-disrupting effects [52], which may potentially influence fatal interactions with fishing activities. Data on Tt and their specific distribution [46] indicate an uneven distribution of fishery interactions across GSAs, highlighting regional variations in both fishing practices and cetacean habitat preferences. For instance, the dominance of Sc in GSA 10 and GSA 19 suggests that these areas may harbor habitats or prey resources preferred by this species, potentially increasing their overlap with fishing activities. Conversely, the significant representation of Tt in GSA 17 and GSA 18 aligns with their known preference for coastal environments in this region, where fishing activities are more intense [51]. These results emphasize that an effective mitigation of fishery interactions requires region-specific management strategies. As previously mentioned regarding overall numbers, the significant decline in the annual stranding rates of Sc with evidence of fishery interaction over the three macro-periods may indicate a potential reduction in anthropogenic pressures and improved management practices. Additionally, this trend could reflect environmental or seasonal changes, as well as ongoing disease outbreaks. However, it should be noted that this shift is likely related to improvements in the analysis of evidence within the tiered approaches over time.

During P2 and P3, fishery interaction was confirmed as the cause of death in 10.32% of examined carcasses, with Tt being disproportionately affected. Data on fishery interaction as COD align with previously reported findings, with a predominance of adult males, likely due to riskier behaviors, again confirming that the opportunistic foraging behavior of Tt on gillnets could also have lethal consequences [48]. No other fishing gear has been identified in this study as a direct threat to cetaceans in Italian waters, besides passive gears mainly used in the Adriatic Sea, and clear evidence of the occasional impact of driftnets, still illegally used in the Tyrrhenian Sea and affecting Pm. The Croatian stranding monitoring similarly highlighted the role of gillnets [25] as a life-threatening gear for Tt. Net fragments have been found entangling the larynx, external appendages, or even as foreign bodies in the stomach [28]. This information validates the relevant impact of gillnets on cetacean populations in the Italian waters, with 56% of COD related to this gear. This represents a higher value compared to data from a global meta-analysis conducted between 1990 and 2020, which reported rates ranging from 2% to 21.8% [53], and to a review of morbidity and mortality in small Odontoceti in the Southern Hemisphere, where gillnets were implicated in 20% of cases [54]. All these reports confirm that gillnets remain a difficult challenge to address and require species and region-based management strategies. In fact, despite frequent reports of intense interactions with trawlers in various areas, including the Adriatic Sea [55,56], data from *post-mortem* investigations appear to support observations from onboard studies that indicate a low rate of lethal interactions [57]. As already stressed above, most of the recognized interactions are probably incidental or related to a specific individual or pod behavior. The persistence of physical injuries such as sharp traumas, or penetrating wounds deliberately caused by fishermen, despite a clear decrease over time, particularly in GSA 10 and GSA 19, an evidence of a shift in the perception of marine workers and the results of best practices [51]. Amputation of the appendages, the use of firearms or sharp objects, suggests that fishermen resent damage to gear, but also that they are still not familiar with proper handling procedures for live animals and carcasses found entangled or by-caught [58,59].

The collection of stranding data over an extended period plays a pivotal role in monitoring the effects of conservation measures, and the present long-term data highlight real issues addressed by an evidence-based approach. For this reason, the implementation of diagnostic and forensic frameworks aimed at evaluating specific phenomena, such as the one developed within the LIFE DELFI project, could yield a better understanding of various challenges and contribute to more effective conservation strategies. A target identification of the different fishing gears in the various areas would be a key factor to promote more successful mitigating measures to be adopted in conservation efforts. A better understanding of the direct impact of different fishing gear could be achieved through “*post-mortem* conservation” in combination with collaborative efforts among Mediterranean countries. Unfortunately, *post-mortem* investigations cannot offer real insight into some indirect effects of fishing activities, such as overfishing, apart from data coming from the NCC evaluation and the stomach content [48]. More targeted and practical actions are needed to investigate and mitigate the impact of common and frequent human activities such as fishing, marine litter, and marine traffic, as well as to establish threshold values in support of conservation regulations. The combination of scientific research with targeted management actions may mitigate the impacts of fisheries on cetaceans and ensure the long-term sustainability of both marine biodiversity and human livelihoods under a One Health perspective.

## Supporting information

S1 Table Evidence of fishery interaction in stranded cetaceans along the Italian coast (1986–2023). Summary of stranded cetaceans examined for signs of interaction with fishing activities. For each individual, year and GSA (Geographical Sub Areas), species, sex (M = Male, F = Female; ND = Not Determined), and age class (a = Adult; j = juvenile; ND = Not Determined) are reported. Evidence of fishery interaction was assessed by noting: by-catch in animal history, presence of fishing gear, larynx entanglement, ingestion of fishing gear, net marks, sharp wounds and appendages amputation, and penetrating wounds. Each parameter is coded as 0 (absent) or 1 (present).
(XLSX)

S2 Table Summary of fishery-related mortality in stranded cetaceans (2015–2023). Summarizes information on the cases of fishery-related mortality. For each individual, the year, GSA (Geographical Sub Areas), species, age class, sex (M = Male, F = Female; ND = Not Determined), and DCC (Decomposition Condition Code) are reported. The Category describes the type of interaction or trauma associated with the fishery, including by-catch (PUE), larynx entanglement, entanglement, and intentional injury. The Fishing gear column indicates the type of fishing gear involved.
(XLSX)
